# Ions, the Movement of Water and the Apoptotic Volume Decrease

**DOI:** 10.3389/fcell.2020.611211

**Published:** 2020-11-25

**Authors:** Carl D. Bortner, John A. Cidlowski

**Affiliations:** Signal Transduction Laboratory, Department of Health and Human Services, National Institute of Environmental Health Sciences, National Institutes of Health, Research Triangle Park, NC, United States

**Keywords:** apoptosis, AVD, RVI, RVD, ion channels, water channels, aquaporins

## Abstract

The movement of water across the cell membrane is a natural biological process that occurs during growth, cell division, and cell death. Many cells are known to regulate changes in their cell volume through inherent compensatory regulatory mechanisms. Cells can sense an increase or decrease in their cell volume, and compensate through mechanisms known as a regulatory volume increase (RVI) or decrease (RVD) response, respectively. The transport of sodium, potassium along with other ions and osmolytes allows the movement of water in and out of the cell. These compensatory volume regulatory mechanisms maintain a cell at near constant volume. A hallmark of the physiological cell death process known as apoptosis is the loss of cell volume or cell shrinkage. This loss of cell volume is in stark contrast to what occurs during the accidental cell death process known as necrosis. During necrosis, cells swell or gain water, eventually resulting in cell lysis. Thus, whether a cell gains or loses water after injury is a defining feature of the specific mode of cell death. Cell shrinkage or the loss of cell volume during apoptosis has been termed apoptotic volume decrease or AVD. Over the years, this distinguishing feature of apoptosis has been largely ignored and thought to be a passive occurrence or simply a consequence of the cell death process. However, studies on AVD have defined an underlying movement of ions that result in not only the loss of cell volume, but also the activation and execution of the apoptotic process. This review explores the role ions play in controlling not only the movement of water, but the regulation of apoptosis. We will focus on what is known about specific ion channels and transporters identified to be involved in AVD, and how the movement of ions and water change the intracellular environment leading to stages of cell shrinkage and associated apoptotic characteristics. Finally, we will discuss these concepts as they apply to different cell types such as neurons, cardiomyocytes, and corneal epithelial cells.

## Introduction

Cell survival depends on maintaining cellular stability from altered environmental conditions that occur from both inside and outside the cell. Cellular stability is accomplished through numerous homeostatic processes which allows cells to self-regulate and/or adjust various biological systems providing an unvarying environment to thrive and flourish. Examples of biological systems that cells maintain include glucose levels, acid-base balance, calcium levels, and fluid volume. Cellular stress results in the activation of a variety of Intracellular mechanisms including the DNA damage response, the unfolded protein response, cell senescence, and regulated cell death (Galluzzi et al., 2018). What drives the homeostatic balance of many biological systems is the movement of monovalent ions that results in a change in water content to alter the concentration of glucose, acids/bases, and calcium. Therefore, ionic fluidity and the movement of water via this mechanism or through specific water channels have a dramatic impact on cell viability. Failure of these homeostatic processes can signal the cell to die. Interestingly, even in death, cells attempt to maintain some sense of biological homeostasis by undergoing a programmed cell death process known as apoptosis. As such, activation of apoptosis is the body’s attempt to remove unwanted or dying cells without affecting neighboring healthy cells.

## Necrosis vs. Apoptosis

Over centuries of scientific discovery, the analysis of dying cells has not been a field of rigorous study. Cells die due to injury, accident damage, or “old age” after fulfilling their purpose to a point where they are no longer needed. Up until the early 1970s, necrosis was the term used to described dying cells defined as an accidental cell death process characterized by cell swelling followed by eventual cell lysis. The release of intracellular products of the dying cell into the extracellular space results in an inflammatory response leading to further damage in the surrounding tissue. As the corpse of a dead cell does not lend one a great deal of substance to explore, attention focused on understanding the inflammatory response, the attraction of leukocytes, and the removal of the dead cell material. However, observations by Kerr (1965, 1970) led to the understanding of a distinctive type of necrosis termed “shrinkage necrosis” (Kerr, 1971). From these early studies, it was evident that the loss of cell volume or cell shrinkage was a distinguishing feature of this controlled cell deletion process. Further study defined this event as a vital, active, and inherently programmed biological process known as apoptosis (Kerr et al., 1972).

There are many discriminating features when comparing necrosis to apoptosis (Nikoletopoulou et al., 2013; D’Arcy, 2019). Necrosis is initiated from external factors that results in detrimental effects on the cell including ATP depletion, cell swelling, membrane disruption, and eventual lysis culminating in an inflammatory response. In contrast, apoptosis is considered a physiological mode of cell death initiated by inherent mechanisms that results in chromatin condensation, cell shrinkage, membrane blebbing leading to the formation of apoptotic bodies that are engulfed by neighboring cells or macrophages. Therefore, apoptosis culminates in a silent process with no noticeable symptoms. Given the distinct cellular events surrounding death by necrosis vs. death by apoptosis, the change in cellular morphology is the most visible characteristic that can easily discriminate between these two diverse modes of cell death.

While necrosis and apoptosis exemplify the extreme modes of cell death, many other cell death processes have been identified and defined. Necroptosis is an inflammatory form of regulated (programmed0 necrotic cell death considered a viral defense mechanism that lacks caspase activation resulting in leakage of the cellular contents into the extracellular space. Similarly, pyroptosis is a highly inflammatory form of programmed cell death that occurs frequently upon infection with intracellular pathogens and is characterized by the formation of the inflammasome (pyrotosome). While caspase-dependent, pyroptosis uses a distinct set of proteolytic enzymes (caspases 1, 4, and 5) then apoptosis and the activation of pore-forming proteins known as geasermins results in water influx and cell membrane rupture. Thus, similar to necroptosis, pyroptosis is not considered immunologically silent. Additionally, ferroptosis is an iron-dependent programmed cell death characterized by the accumulation of lipid peroxides triggered by the failure of the glutathione-dependent antioxidant defense mechanism. Cells undergoing ferroptosis typically contract, then swell, releasing their intracellular contents. The most similar mode of cell death that mimics apoptosis is the death of red blood cells known as eryptosis. Insults such as hyperosmolarity, oxidative stress, and heavy metal exposure can result in erythrocytes undergoing cell death characterized by cell shrinkage, membrane blebbing, activation of proteases, and externalization of phosphatidylserine. A comparison of the characteristics that define these modes of cell death is shown in Table 1.

**TABLE 1:** Characteristics of various regulated modes of cell death.

Characteristic	Apoptosis	Necrosis	Necroptosis	Pyroptosis	Ferroptosis	Eryptosis
Cell shrinkage	Yes	No	No	No	No	Yes
Cell swelling	No	Yes	Yes	Yes	No	No
Nucleus fragmentation	No	No	No	Yes	No	No
Membrane blebbing	Yes	No	Yes	Yes	No	Yes
Caspase activation	Yes	No	No	Yes	No	Yes
DNA fragmentation	Yes	No	No	Yes	No	No
Cell lysis	No	Yes	Yes	Yes	no	No
Inflammation	No	Yes	Yes	Yes	Yes	Yes
Regulated	Yes	No	Yes	Yes	Yes	Yes

In total, there have been 34 different modes of cell death described in the literature (Liu et al., 2018b). This includes the orderly degradation and recycling of cellular components known as autophagy; an ischemic cell death resulting from ATP depletion known as oncosis; death of anchorage-dependent cells that detach from the surrounding extracellular matrix known as anoikis; and a programmed mode of necrotic cell death in fibroblasts known as nemosis. While many of these modes of cell death are similar in nature, they can provide a unique characterization of the physiology in a clinical or pathological setting. For example, mitotic catastrophe that occurs due to premature or inappropriate entry of cells into mitosis is the most common mode of cell death in cancer cells exposed to various chemotherapeutic treatments. Thus, the use of mitotic catastrophe has a very relevant connotation in this clinical setting. In this review, we will focus on the classical physiological mode of cell death, apoptosis; and examine cell death in several cell type model systems in regards to ion and water movement that results in the loss of cell volume.

## Maintaining Fluid Volume Homeostasis

Alterations in cell morphology are key in distinguishing necrosis and apoptosis, thus variations in cellular water content must occur suggesting that maintaining water balance is critical for cell survival. Thus, inherent cellular mechanisms have developed to combat changes in the extracellular environment that impacts a cells hydration state. In general, a sudden change in solute concentration surrounding a cell results in an osmotic stress, also described as an osmotic shock. When the extracellular solute concentration is low (hypo-osmotic stress), cells can rapidly gain water. In contrast, when the extracellular solute concentration is high (hyper-osmotic stress), a rapid loss of water occurs from cells. Simply noted, water will flow in the direction of higher solute concentration, signifying solute flux as a central determinant of water movement. As cells have a defined perimeter and limited capacity to either contract or expand, most cells respond to changes in these environmental conditions with rapid ionic fluxes that alter their intracellular environment to adjust to the change in the extracellular environment (Lang et al., 1998; Hoffmann and Pedersen, 2011; Pasantes-Morales, 2016; Delpire and Gagnon, 2018).

The gain in water that occurs when cells encounter a hypo-osmotic environment (a decrease in external osmolarity) is immediately countered with an active recovery process known as regulatory volume decrease (RVD). This inherent adaptation process involves the flux of ions, mainly potassium and chloride, along with various organic osmolytes from the cell (Hoffmann, 2000; Yang et al., 2012). Potassium is the most abundant monovalent ion in the cell and permeates from the cell through various channels including voltage-gated, Ca^2++^-activated, inwardly rectifying, and two-pore-domain potassium channels (Pasantes-Morales, 2016). The precise potassium channels activated during RVD appears to be both cell-type and stimulus-specific. Along with potassium channels, voltage-sensitive chloride channels also have a key role in RVD in maintaining an overall electrically neutral ionic state. The voltage-regulated anion channel (VRAC) has been a channel of intense interest as VRAC is also permeate to large molecules such as gluconate and glutamate that can further facilitate a restoration in cell volume (Pedersen et al., 2016).

Cells encountering a hyper-osmotic environment (an increase in external osmolarity) immediately shrink and activate a regulatory volume increase (RVI). During this process, various mechanisms are activated to increase the concentration of intracellular osmolytes. Sodium is the most abundant ion outside the cell, and sodium enters the cell through various electroneutral cotransporters and exchangers including the Na^+^–Cl^–^ cotransporter (NCC), the Na^+^/K^+^/2Cl^–^ cotransporter (NKCC), and the Na^+^–H^+^ exchanger coupled to the Cl^–^/HCO_3_^–^ exchanger (Pasantes-Morales, 2016). Additionally, the initial increase in intracellular sodium that occurs during RVI is alleviated through the activation of the Na^+^–K^+^-ATPase, that resets the initial intracellular ionic environment. Similar to the activation of RVD, the precise transporters and exchangers activated during RVI is not completely understood and occurs in both a cell-type and stimulus-specific manner.

The importance of inherent volume regulatory mechanisms in protecting cells from adverse changes in the extracellular environment was illustrated when T-cells, that lack a normal RVI, were subject to hypertonic stress (Bortner and Cidlowski, 1996). In the absence of an inherent RVI, these T-cells shrank and underwent a rapid and systematic cell death that was shown to be apoptosis, while cells such as COS-7, L-cells, and PC12 cells, that can regulate their volume via RIV, were resistant to hypertonic-induced stress and survived. Increased tonicity augmented serum-deprived induced apoptosis in vascular smooth muscle cells (Orlov et al., 1996). In support of the concept of volume regulatory mechanisms protecting cells from death, inhibition of hypertonicity-induced cation channels was shown to sensitize HeLa cells to undergo apoptosis when the extracellular osmolality was increased (Shimizu et al., 2006). Interestingly, while hyperosmolarity did not initially induce apoptosis in rat hepatocytes that respond with an RVI, Reinehr et al. (2002) showed this condition did target the CD95 receptor to the plasma membrane sensitizing the cells to Fas ligand-induced apoptosis. While in the clinical setting, mannitol therapy has been widely used for acute and subacute reduction in brain edemas resulting from closed-head trauma, and ischemic brain swelling to improve cerebral blood flow, Malek et al. (1998) pointed out potential deleterious effect of hyperosmotic treatment on the vascular endothelium due to mannitol’s ability to induce apoptosis. This suggests caution should be exercised for the clinical use of osmotic diuretics such as mannitol to avoid detrimental and often lethal effects to surrounding cells and tissue.

## The Advent of AVD

A defining characteristic of cell death as outlined earlier is a change in cell volume. This simple and straightforward visual cue allows one to immediately categorize the two most common modes of cell death that has or is occurring. The loss of cell volume that occurs during apoptosis and gain in cell volume as observed during necrosis both occur in the absence of osmotic changes in the extracellular environment. Consequently, the term necrotic volume increase (NVI) was proposed to describe the influx of sodium, lactate, and other osmolytes into cells leading to cell swelling during this accidental or necrotic cell death process (Barros et al., 2001). NVI is thought to be due in part to a dysregulation of the inherent RVD, specifically an impairment of volume-sensitive Cl^–^ channels (Okada et al., 2004, 2019) and subsequently, an acid-sensitive outwardly rectifying (ASOR) anion channel (Wang et al., 2007).

In contrast, the term apoptotic volume decrease (AVD) has been applied to describe the loss of cell volume or cell shrinkage during the physiological or apoptotic cell death process (Maeno et al., 2000; Okada et al., 2004). What became apparent from early studies investigating the loss of cell volume during apoptosis was that AVD most likely was not a novel volume mechanism, but occurred via sharing or commandeering inherent RVD channels/transporters for a new purpose during the programmed process (Okada et al., 2001, 2004; Okada and Maeno, 2001). Of particular note is VRAC; the volume-activated anion channel that is essential to the apoptotic death machinery. VRAC is activated by a change in intracellular ionic strength, increased intracellular calcium, ROS, and phosphorylation (Kunzelmann, 2016), however a complete understanding these signaling cascades is not known. The exact nature of this channel remained unknown until the recent identification of LRRC8 proteins as a key component of VRAC (Qiu et al., 2014; Voss et al., 2014). While LRRC8 isoforms have been shown to contribute to RVD and sense changes in ionic strength (Syeda et al., 2016), it has been suggested that this volume-regulated anion channel may not be essential for AVD (Planells-Cases et al., 2015; Jentsch, 2016; Sirianant et al., 2016). Moreover, it is important to note that the reprogramming of RVD to AVD during apoptosis must likely involves the inactivation of RVI that would normally compensate for a loss of cell volume.

As ion flux was known to underlie cell volume regulatory processes, ion channels became a central focus for AVD. Two-pore domain K^+^ (K(2P)) channels were suggested to underlie potassium efflux during AVD in mouse embryos (Trimarchi et al., 2002). AVD was shown to be accelerated upon staurosporine-induced apoptosis of COS-7 and pulmonary artery smooth muscle cells (PASMC) overexpressing a delayed-rectifier voltage-gated K^+^ channel (Brevnova et al., 2004). Studies using a calcium-induced apoptosis lymphocyte/thymocyte model showed that AVD could be blocked with inhibitors of IKCa1, preventing the externalization of phosphatidylserine and cell death (Elliott and Higgins, 2003). In endothelial cells challenged with staurosporine, AVD was inhibited with the chloride channel blocker phloretin, again preserving cell viability (Porcelli et al., 2004).

Early X-ray microanalysis showed an increase in intracellular sodium coupled with a decrease in intracellular potassium within 3 h of oxidized low-density lipoprotein exposure in monocyte-macrophages (Skepper et al., 1999). Subsequent X-ray microanalysis studies confirmed a two-phase change in intracellular using staurosporine-treated U937 cells (Arrebola et al., 2005a,b, 2006). These authors showed during late stage apoptosis the potassium concentration continued to decrease, while chloride increased along with an increase in sodium. In a follow-up study, this group also showed that the initial stage of apoptosis in UV-induced U937 cells were characterized by a decrease in potassium and chloride, with the largest decrease occurring from the mitochondria (Arrebola et al., 2006). An earlier study by Bortner and Cidlowski (2003) had showed an increase in intracellular sodium in anti-Fas treated Jurket cells, that was in part to be due to the inhibition of the Na^+^/K^+^-ATPase, as direct inhibition of this ionic pump with ouabain enhanced apoptosis. This study also that cell shrinkage could be uncoupled from apoptosis, which was later confirmed in a study comparing staurosporine- and etoposide-induced apoptosis in U937 cells (Yurinskaya et al., 2005a).

Additionally, several other studies also eluded to an increase in intracellular sodium that accompanied the initial loss of intracellular potassium during AVD (Jonas et al., 1994; Offen et al., 1995; Fernandez-Segura et al., 1999; Yurinskaya et al., 2005b). What was clear from these early studies on AVD was while no single ionic channel or pathway could account for the loss of cell volume during apoptosis, the movement of ions was a critical part of the cell death process. More recent studies have solidified this relationship between AVD and ion flux, as a computational study on the redistribution of ions and water underlying AVD in staurosporine treated human lymphoma cells (U937) concluded that along with a significant increase in chloride and potassium permeability coupled with a decreased permeability of sodium, there was also the progressive decrease in the Na^+^/K^+^ activity (Yurinskaya et al., 2019).

In some model systems, it has been suggested that apoptosis can occur in the absence of a loss of cell volume. A modest decrease in cell volume in serum-deprived vascular smooth muscle cells does not trigger the apoptotic machinery (Orlov et al., 2004). Interestingly, Nolin et al. (2016) reported swelling of the whole cell prior to its entry into apoptosis; a phenomenon also noted in an earlier study using time-lapse, dual-image surface reconstruction of staurosporine treated vascular smooth muscle cells (Platonova et al., 2012). These studies illustrate that more refined changes can occur during AVD, that typically may go unnoticed in many apoptotic model systems.

## Stages of AVD

Following initial studies describing various ion flux pathways and transporters that were involved in and defined AVD, the overall nature of this process was examined which focused on both water and ion movement. Radiation-induced changes in cell size of rat thymocytes was shown to occur in two distinct stages (Klassen et al., 1993). An early reversal of intracellular ions was observed which defined an initial or primary stage of apoptosis in lymphocytes (Bortner et al., 2008). Of interest was this primary stage of AVD occurred during both intrinsic and extrinsic apoptosis. During the primary stage of AVD, an increase in intracellular sodium, coupled to a decrease in intracellular potassium occurred that resulted in a 20–40% decrease in cell volume. It was hypothesized that this reversal of intracellular ions was the cell’s attempt to compensate for the loss of one ion (potassium) for another (sodium), however, in total, an overall decrease in cell volume occurred. During the secondary stage of AVD, both intracellular sodium and potassium were lost resulting in an 80–85% decrease in cell volume. Additionally, this secondary stage was shown to be prevented upon disruption of the actin cytoskeleton (Bortner et al., 2008). Poulsen et al. (2010) described three distinct stages of AVD in cisplatin-induced Ehrlich ascites tumor cells; an early, transitional, and secondary stage. The early and secondary stages were defined with a loss of ions, specifically potassium, sodium, and chloride, that resulted in a 30% loss of water (early stage), and a further reduction in water in the secondary stage. The transitional stage was defined solely with an increase in sodium and chloride. Interestingly in both aforementioned studies, the increase in intracellular sodium suggests a counter or protective response even as a cell is dying. Interestingly, a protective response was illustrated by Yurinskaya et al. (2012) where U937 cells under hypertonic stress initially responded with an RVI prior to AVD. Whether RVI and AVD are independently activated and AVD is observed only apparent at a later time; or RVI becomes inhibited or fails signaling AVD is unclear.

The use of live fluorescence and transmission-through-dye microscopy showed two morphologically distinct stages of volume changes (Kasim et al., 2013). Using actinomycin-D treated HeLa cells, the first stage was defined by extensive blebbing with a temporary volume increase, while the second stage showed a 40% decrease in cell volume and was considered to represent AVD. Interestingly in this model system, both stages had an increase in intracellular sodium, which again suggests a protective cellular response even in the act of dying. In a latter study, correlative light and cryo-scanning transmission electron microscopy (cryo-STEM) was employed to study stage-specific changes in water and ion movement in actinomycin D treated HeLa cells (Nolin et al., 2016). This technology allowed the authors to observe changes of water and ions not only in the cell as a whole, but also in various cellular compartments. Overall, the authors observed a loss of potassium throughout the entire cell death process. Since previous studies suggested an early increase in sodium and loss of chloride, followed by a decrease in sodium coupled to a further decrease in chloride, the latter appeared to be restricted to the mitochondria. Finally, during the late stage of cell death, an increase in sodium and chloride was noted that may ensue due to a loss of membrane integrity. Overall, these studies illustrate a complex and multifaceted nature of volume dynamics during cell death.

Many studies, including the one outlined above, have shown that the concept of AVD or cell shrinkage during apoptosis is not a simple and straight-forward process. Of particular note is the idea that AVD is distinct and independent from the loss of cell volume that occurs upon separation of apoptotic bodies. Overall, the volume dynamics that reflect AVD is a direct consequence of ion flux. The observation of whether a cell shrinks or not during the cell death process appears to be both cell-type and stimulus specific (Yurinskaya et al., 2005a; Orlov et al., 2013). Therefore, water content of non-apoptotic and apoptotic cells may not be of much consequence, as it is not the change in cell volume that is critical, but the flux of ions that has a greater impact on the cell death program.

## The Relevance of AVD to Other Apoptotic Events

Prior to AVD becoming an acknowledged scientific concept, intracellular ions were known to play a critical role in water loss during apoptosis (McCarthy and Cotter, 1997; Yu et al., 1997, 1999a). Furthermore, and as mentioned above, many studies support the idea of ion flux having a critical role in the apoptotic program, with the change in cell volume a by-product of this ion movement. However, the question remained as to what consequence these ion fluxes have on apoptosis? Early it was shown that DNA degradation during apoptosis in anti-Fas treated Jurkat cells correlated with the shrunken population of cells, and inhibition of cell shrinkage via high extracellular potassium prevented this characteristic (Bortner et al., 1997). Interestingly, anti-Fas treatment of Jurkat cells initially placed in hypotonic medium to swell and activate an RVD, thus lowering the intracellular potassium and chloride concentration, resulted in enhanced cell death, suggesting that a change in ions was having a greater effect on the cell death program than the actual change in cell size. In a follow-up study, normal intracellular levels of potassium were shown to inhibit both apoptotic DNA fragmentation and caspase-3 activation, however, once the caspase was activated, the level of intracellular potassium had no consequence (Hughes et al., 1997). An early study examining calcium signaling during apoptosis showed that anti-Fas treated Jurkat cells under complete calcium-free conditions resulted in only the inhibition of DNA fragmentation, suggesting nuclear activity was the only component of the apoptotic machinery that was sensitive to changes in intracellular calcium (Scoltock et al., 2000). Additionally, it was shown that elevated extracellular potassium prevented phosphatidylserine externalization, mitochondrial depolarization, and cytochrome c release, along with caspase activation upon both chemical and death receptor induced apoptosis (Thompson et al., 2001). This same group also showed that physiological concentrations of potassium inhibited cytochrome c-dependent apoptosome formation, thus preventing the activation of caspase-9 (Cain et al., 2001). In a more recent study, Tanaka et al. (2015) examined the relationship between AVD and translocation of phosphatidylserine on the cell surface and membrane blebbing using time-lapsed imaging coupled with scanning ion conductance microscopy. Here, the authors using staurosporine-treated neurons reported that the loss of cell volume occurred prior to the externalization of phosphatidylserine and membrane blebbing, suggesting that these morphological events may be independent of AVD and the concurrent ion flux during apoptosis.

Since our initial understanding of the important role ion fluxes play in regulating the apoptotic machinery, considerable attention has focused on the specific channels and transports involved in this process, that may or may not contribute to AVD. In an early study using human leukemia cells (HL-60), apoptotic change in intracellular ions was prevented upon inhibition of the Na^+^, K^+^-ATPase pump or the Ca^2+^-dependent K^+^ channel (McCarthy and Cotter, 1997). Caspase-dependent stimulation of voltage-gated potassium (Kv1.3) channels was shown in Fas-ligand treated Jurkat cells to result in potassium efflux, cell shrinkage, and apoptosis (Storey et al., 2003). While potassium is a major intracellular ion whose loss would result in a reduction of cell volume, it is not the only ion to consider in understanding the relevance of AVD. The early and rapid increase in intracellular sodium shown to occur during anti-Fas induced apoptosis resulted in a depolarization of the plasma membrane (Bortner et al., 2001). The depolarization of the plasma membrane had previously been shown to occur in part via the inhibition of the Na^+^/K^+^-ATPase (Bortner et al., 2001; Mann et al., 2001). Thus, over the past 2 plus decades, many studies have solidified the critical role for ion flux in regulating the apoptosis process (reviewed in Lang and Hoffmann, 2012; Kondratskyi et al., 2015).

Conversely, Börjesson et al. (2011) suggested that a decrease in intracellular potassium concentration is not obligatory for apoptosis. In an oocyte model treated with staurosporine, a loss of intracellular potassium was observed along with the activation of caspase-3. Interestingly, when oocytes densely expressed Shaker voltage-gated potassium channels, a loss of potassium was not observed, implying that the dense Kv channel expression makes oocytes resistant to apoptosis. However, caspase-3 activity was still observed. While the authors concluded that a decrease in intracellular potassium concentration is not required for apoptosis, other ions known to have a role in decreasing the overall ionic strength was not explored. Largely, these afore-mentioned studies illustrated the importance of ions in the programmed cell death process and suggested that an overall decrease in intracellular ionic strength permits the activation of the apoptotic machinery.

## Linking Apoptosis and Water Movement: Aquaporins

Water channels, or selective water pores known as aquaporins (AQPs) play a critical role in mediating cellular water flow, and are crucial for the regulation of water homeostasis. Aquaporins provide a mechanism for the rapid movement of water across diverse membranes having a major regulatory effect in regard to changes in cell volume (Day et al., 2014). In the early 2000’s, Krane et al. (2001) reported that salivary acinar cells deficient in aquaporin 5 (AQP5) had a decrease in water permeability in response to hypertonicity-induced cell shrinkage and hypotonicity-induced cell swelling. While it is unclear exactly how aquaporins regulate this movement of water, it has been proposed that a critical factor is the number of channels expressed on the cell membrane. AQP5 was shown to have a dose-responsive decrease in response to a hypotonic stimulus (Sidhaye et al., 2006). This study also showed that inhibition of the cation channel transient receptor potential vanilloid (TRPV) 4 prevented the reduction of AQP5. In a latter study, a rapid translocation of AQP1 was shown to occur under hypotonic conditions coupled with an increase in intracellular calcium (Conner et al., 2012). As these studies involved different aquaporins, both studies indicated that alterations in calcium were required for the change or translocation of the channels while illustrating their response in the regulatory volume response. Additionally, these studies indicate that ions, particularly calcium, may have an important regulatory role in water channel function, as opposed to regulation at the level of the channel itself. Furthermore, while initially thought to facilitate only the movement of water, it is now understood that aquaporins can also permeate other small solutes such as anions, urea, and glycerol, that would also have a role in the volume regulatory response.

Aquaporins have been shown to play a vital role during cell death as inhibition of aquaporin 1 (APQ1) was shown to prevent AVD and the subsequent downstream apoptotic events such as cell shrinkage, mitochondrial membrane permeability, caspase 3 activation, and DNA degradation (Jablonski et al., 2004). Additionally, decreased expression of APQ8 and APQ9 correlated with the lack of water movement in apoptotic-resistant tumor cells (Jablonski et al., 2006). Overexpression of AQP3 and AQP9 in human melanoma cells significantly increase the chemoresistance of these cells to arsenite via down-regulation of p53 and up-regulation of Bcl-2 and XIAP (Gao et al., 2012). Thus, like the overall mechanism of apoptosis, the role aquaporins play during cell death process appears to be cell-type and stimulus specific.

Since the mid 1990’s, an increasing number of studies have focused on aquaporins and the movement of water, with many focusing on specific physiological systems or pathological conditions (Tait et al., 2008; Zeleina, 2010; Ma et al., 2011; Schey et al., 2014; Tie et al., 2017). For example, in the central nervous system, aquaporin 4 (AQP4) is noted as the main water channel. Lactacystin (proteosomal-inhibition) induced apoptosis in cortical neurons showed AQP4 was highly downregulated suggesting other AQPs may be involved during programmed cell death (Chen et al., 2008). Interestingly, this study showed that APQ8 and APQ9 were highly upregulated upon lactacystin treatment. However, when staurosporine was used to induce apoptosis, both APQ8 and APQ9 were down regulated, suggesting aquaporins expression and function during apoptosis is stimulus specific. In a myocardial infarction model, cardiomyocytes deficient in AQP1 showed a reduced level of apoptosis, suggesting AQP1 has a positive role in the execution of the cell death process (Li et al., 2015). And as early as 2004, aquaporins, specifically AQ1 was shown to be decreased in human cornea endothelial disease, and in mouse corneas subjected to corneal endothelial injury (Macnamara et al., 2004). Thus, as is the case for ion channels and transporters, aquaporins are expressed in a range of cells and show a variety of roles in regards to apoptosis.

## Ion Movement and AVD in Unique Model Systems

Lymphoid cells have been a favorite model system to study AVD and apoptosis. However, over the past decade, other cell type such as neuronal cells, cardiomyocytes, and corneal epithelial cells have been increasingly studied due to their unique characteristics and prevalence in various human diseases. While the brain as a whole shows an RVI upon hypertonic perturbations, cultured and acutely isolated neurons do not. Similarly, reports of RVI in cardiomyocytes are limited to murine atrial cardiac myocyte cultured cell lines such as HL-1 (Cacace et al., 2014). As both neurons and cardiomyocytes have an RVD, it is of interest to determine their relationship to AVD upon apoptosis. While corneal epithelial cells can regulate their cell volume under both hypertonic or hypotonic conditions, the cornea is shielded by a protective tear film that makes this a model of interest in understanding ion flux and fluid movement upon cell death.

## Neuronal Cells and Cell Death

Neuronal cells offer an attractive model to study the movement of water, ions and apoptosis. Early on it was shown that mouse neocortical neurons treated with staurosporine or serum deprivation resulted in an early enhancement of delayed rectifier (IK) currents and a loss of intracellular potassium resulting in apoptosis (Yu et al., 1997). Apoptosis was reduced upon addition of the potassium channel blocker TEA or elevated extracellular potassium. In turn, exposure of neuronal cells to the K^+^ ionophore valinomycin or the K^+^-channel opener cromakalin induced apoptosis, suggesting that neuronal cell death may follow a similar series of ion flux as observed in more classical model systems. Shortly after this, NMDA receptor-mediated potassium flux was shown to contribute to neuronal apoptosis during brain ischemia (Yu et al., 1999a), signifying an expansion of ion flux beyond classical voltage-gated ion channels. In a second study from this group, inhibition of potassium channels with clofilium attenuated C2-ceramide induced neuronal apoptosis (Yu et al., 1999b), as well as hypoxia- and ischemia-induced neuronal death, both *in vitro* and *in vivo* (Wei et al., 2003). These early studies illustrating the critical role for potassium during neuronal cell death set the stage for further scientific investigation of neuronal cell death.

**FIGURE 1:**
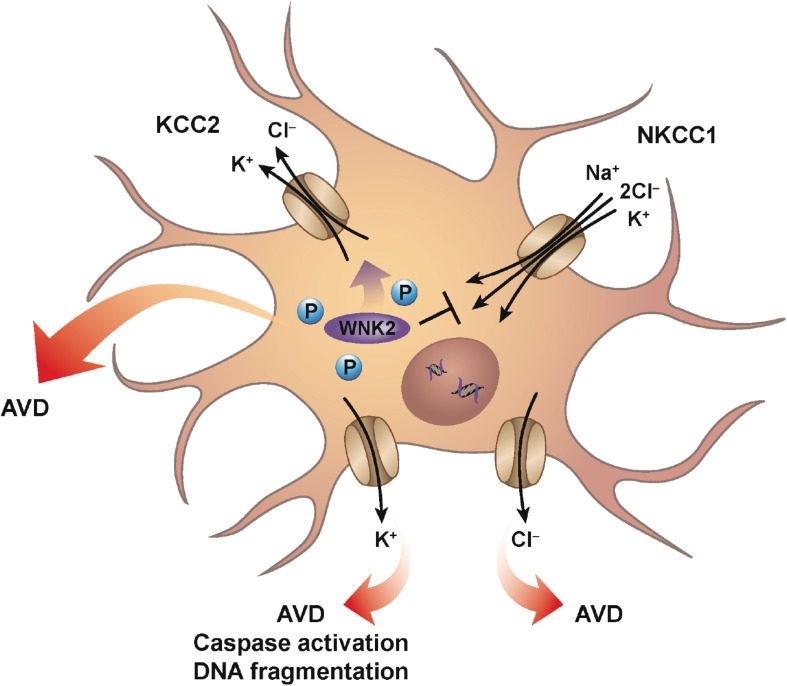
Neuronal AVD. Mechanisms similar for classical RVD are engaged during neuronal AVD. Ionic cotransporters and cotransporters, mainly involving the flux of chloride are activated to counter the imbalance of intracellular water due to hypotonic conditions. For example, conventional ionic transport mechanisms such as NKCC1 and KCC2 are oppositely-regulated via serine–threonine phosphorylation such that dephosphorylation results in the inhibition of NKCC1, while simultaneously activating KCC2. The net result is the loss of both intracellular potassium and chloride with the parallel decrease in water. Additionally, individual potassium and chloride channels have also been shown to have a role during neuronal AVD. Interestingly, potassium channel activation was shown to result in AVD, caspase activation, and DNA fragmentation, while chloride channel activation resulted in only AVD.

Neurons, like every other cell in the body, can also be subjected to changes in their extracellular environment. Upon encountering a condition of decreased osmolality, neurons will undergo RVD to achieve a homeostatic balance of water and ions. This RVD occurs via classical ionic channels and transport mechanisms similar to other cell types, and is observed in many neuronal cells including peripheral sympathetic neurons, cerebellar granular cells, along with numerous neuronal cultured cell lines (Wilson and Mongin, 2018). It was suggested that AVD in neurons appears to occur by similar ionic mechanisms to those activated during hypoosmotic-induced RVD (Pasantes-Morales and Tuz, 2006). Cation-chloride cotransporters (CCC) such as the chloride-importing Na–K–2Cl cotransporter (NKCC1) and the chloride-exporting potassium–chloride cotransporter (KCC2) have a significant role in the regulation of neuronal cell volume, along with their role in neurotransmission in the nervous system. These transporters are oppositely regulated via serine–threonine phosphorylation that inhibits NKCC1, but activates KCC2, upon dephosphorylation possibly through the WNK2 kinase (Gamba, 2005; Rinehart et al., 2011; Figure 1). The dephosphorylation of these transporters promotes the efflux of ions, specifically potassium and chloride from the cell resulting in loss of water. Interestingly, numerous studies involving neurons (both primary and cultured) failed to demonstrate a classical RVI response upon hyperosmotic exposure. Additionally, a lack of RVI was also observed in most studies involving cultured astrocytes (reviewed in Wilson and Mongin, 2018). A sound hypothesis for the absence of RVI in various neuronal cells has yet to be proposed, although it has been suggested that cultured neuronal cells may not have the required transmembrane ionic gradients that favor RVI.

During development, proper formation of synapses between neurons in the brain is known to occur via apoptosis. Furthermore, apoptosis is also the most common mode of cell death in various neurodegenerative diseases with increased apoptosis associated with Alzheimer’s and Parkinson’s disease (Yuan and Yankner, 2000), suggesting that apoptosis plays a variety of roles within the central nervous system. As mentioned earlier, and similar to other apoptotic model systems, the loss of intracellular potassium is known to have a critical impact in coordinating the cell death program. Early studies showing the enhancement of delayed rectifier (IK) potassium currents during apoptosis in neocortical neurons, were followed by studies on cultured cortical neurons treated with a variety of apoptotic inducers that showed the involvement of ion flux. Inhibition of chloride channels prevented cell shrinkage, but had no significant effect on caspase activation or DNA fragmentation (Wei et al., 2004; Figure 1). In contrast to chloride channel inhibition, inhibition of potassium channels prevented cell shrinkage, caspase activation, and DNA fragmentation (Figure 1). This study suggests that potassium and chloride have distinct roles during apoptosis, with inhibition of potassium flux exhibiting a greater neuroprotective effect. Furthermore, studies such as these highlight the critical role potassium plays in regulating the apoptotic machinery, again in line with more classical apoptotic model systems.

Over the years, numerous potassium channels have been identified in neuronal cells that have a function during apoptosis including voltage-gated K^+^ channels, inwardly rectifying K^+^ channels, and background channels such as tandem pore domain TWIK and TASK (Chen et al., 2008). Many of these studies have relied on channel inhibition to show their contribution in the cell death process. Of the various potassium channels identified, the delayed rectifier current mediated by Kv2.1 channel has a critical role in apoptogentic potassium efflux in several types of neuronal cells including cortical, nigral, and hippocampal neurons. Pal et al. (2003, 2006) showed that potassium efflux during neuronal cell death involved newly inserted Kv2.1 channels into the cell membrane. As Kv2.1 channels are known to form clusters in the soma and proximal dendrites, Justice et al. (2017) discovered that disruption of these clusters prevented the apoptogenic increase in potassium currents, thus increasing neuronal viability upon exposure to oxidative stress. Recently in primary neurons, Kv2.1 was shown to be substrate for the aspartyl protease BACE2, and upon channel cleavage prevented the outward potassium efflux resulting in reduced apoptosis (Liu et al., 2018a). Furthermore, methamphetamine results in pro-apoptotic effects in primary hippocampal neurons that are abrogated upon inhibition or knockdown of Kv2.1 (Zhu et al., 2018b), suggesting potassium channel inactivation without the use of drugs that specifically block the channel can regulate the apoptotic program. Additionally, this study showed that p38 mitogen-activated protein kinase (MAPK) was also involved as inhibition of this kinase attenuated methamphetamine-induced pro-apoptotic effects and the upregulation of Kv2.1. Moreover, phosphorylation of what are considered pro-apoptotic residues on the N- and C-terminus of Kv2.1 via Src and p38 enhanced the insertion of this potassium channel in the plasma membrane, thus increasing the loss of intracellular potassium during apoptosis (He et al., 2015).

Like many other apoptotic model systems, potassium has been the cationic ion of emphasis, however, as mentioned earlier sodium also has a criterial role in the cell death program, and has been an ion of focus of numerous studies involving neuronal apoptosis. Sun et al. (2020) showed flufenamic acid and mefenaminc acid were neuroprotective by inhibiting voltage-gated sodium channels in a glutamate-induced apoptotic model using neuroblast-like SH-SY5Y cells. In a model resembling epilepsy, hippocampal neurons from rats injected with kainic acid (an NMDA type glutamate receptor agonist) underwent apoptosis that was attenuated upon inclusion of various voltage-gated sodium channel blockers (Das et al., 2010). An increase in intracellular sodium following abnormal hyperexcitation can result in death of neurons, and a recent study probing the mechanism of this model suggested this death occurs via sodium accumulation and/or concomitant potassium loss that impairs mitochondrial function (Kawasaki et al., 2019). Genistein, a primary isoflavone found in soybeans, was shown to inhibit cell death in an *in vitro* model of primary neurons under hypoxic-ischemia (oxygen–glucose deprivation) conditions in part by reversing the classic increase in potassium efflux and decreasing the sodium influx (Ma et al., 2016). Additionally, this group in a latter study employing a different hypoxic-ischemia model (sodium dithionite and glucose deprivation) in cultured rat primary neurons showed that a specific zinc-chelator, TPEN, suppressed apoptosis in primary neurons (Zhang et al., 2017), however whether this was a direct inhibition of ion channels or a change in channel function due to reduced levels of zinc was not determined.

Potassium channel activation is not always congruent with triggering of apoptosis, especially in neurons. Interestingly, potassium channels are common targets for neuroprotective molecules. Activation of mitochondrial ATP-sensitive potassium channels (mKATP) prevented neuronal cell death after ischemia in neonatal rats (Rajapakse et al., 2002), essentially mimicking the protective effects mediated by the preconditioning phenomenon. In an oxygen–glucose deprivation model of cell death in primary rat cortical neurons, diazoxide, a potassium channel activator attenuated neuronal cell death (Kis et al., 2003). Of interest in this study was that a mitochondrial ATP-sensitive potassium blocker (5-hydroxydecanoate) abolished this protective effect, while a non-selective KATP channel blocker (glibenclamide) did not, suggesting the reliance of mKATP for this resistance. Since this early deduction of neuroprotection via opening of mKATP, numerous studies have expanded on this discovery. Neuroprotection via mitochondrial ATP-sensitive potassium channels enhance cell survival against oxidative stress (Yang et al., 2016). In a chronic morphine (CM) preconditioning study of ischemia/reperfusion hippocampal CA1 neurons, inhibition of mKATP channels with 5-hydroxydecanoate (5-HD) significantly increased apoptosis, suggesting that CM preconditioning obstructs apoptosis via activation of mKATP channels (Arabian et al., 2018). Various studies have suggested that the protection afforded by the activation of potassium channels may be due to membrane potential hyperpolarization, and/or increased repolarization speed effectively reducing the level of calcium entry and ATP consumption; both considered pro-apoptotic events. Thus, pharmacological modulation of mKATP has become a promising new therapeutic approach for the treatment of neurodegenerative diseases such as Alzheimer’s (Virgili et al., 2013), along with the treatment of cardiovascular and various oncological diseases (Citi et al., 2018).

## Cardiomyocytes and AVD

**FIGURE 2:**
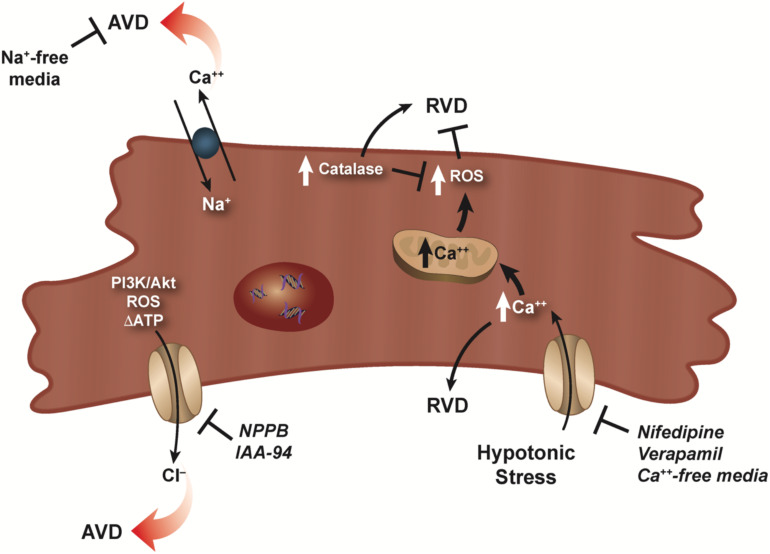
RVD and AVD in cardiomyocytes. Dual role of calcium for RVD in cardiomyocytes is illustrated as a hypotonic-induced increase in intracellular calcium activates RVD. However, a simultaneous increase in ROS masks and/or prevents RVD. RVD can be restored via overexpression of catalase which lowers the ROS concentration. Activation of volume-sensitive chloride channels via kinases, ROS, and/or changes in the level of ATP were shown to have a major effect on cardiomyocyte AVD, which can be prevented upon addition of specific chloride channel blockers. Additionally, reverse mode of the Na–Ca exchanger can also result in AVD as sodium-free conditions prevents this loss of cell volume.

While cardiomyocytes are known to respond to hypoosmotic stress with an RVD, the presence of an RVI under hyperosmotic stress has not been well documented in these cells. It has been shown that HL-1 cardiac myocytes can regulate their volume to hypertonic stress with a classical RVI (Cacace et al., 2014), however, at present, no study has documented the presence of an RVI in primary cardiomyocytes. In the absence of this inherent RVI response, cardiac myocytes respond with a rapid and robust apoptotic response upon hyperosmotic stress (Morales et al., 2000; Galvez et al., 2001). Maldonado et al. (2005) showed that this rapid and pronounced apoptosis in response to hyperosmotic stress in cardiomyocytes could be attenuated by treatment with insulin-like growth factor (IGF-1), setting in motion a series of calcium-related phosphorylation events. More recently, hyperosmotic stress was shown to induce cell death in adult rat cardiomyocytes via mechanism promoting endoplasmic reticulum stress (ERS; Burgos et al., 2019). In this study, cardiomyocytes placed in a hyperosmotic environment resulted in increased expression of various ERS markers, along with an increase in caspase-3 expression and the loss of cell viability. Furthermore, 4-BPA (an inhibitor of ERS), chelating calcium using EGTA, and inhibition of CaMKII prevented hyperosmotic-induced cell death. Interestingly, Zhu et al. (2018a) suggest that in the absence of the protective nature of an RVI response, cardiomyocytes invoke an autophagic pathway to provide a cardioprotective strategy in response to hyperosmotic stress. The complex nature of volume regulatory process in cardiomyocytes was illustrated by Rojas-Rivera et al. (2009). These authors showed that hypo-osmotic stress-induced increase in intracellular calcium, thus activating RVD, however, this also resulted in an increase ROS (Figure 2). This masking of RVD via the increased ROS resulted in necrotic blebs and cell death. Interestingly, the overexpression of catalase, lowered ROS content, and restored RVD (Figure 2).

In the mid 1990s, studies investigating ischemia reperfusion injury showed that cell death of cardiomyocytes occurred predominantly through the programmed cell death process or apoptosis (Cheng et al., 1996; Kajstura et al., 1996). Furthermore, myocardial ischemia can result in osmotic stress on cardiomyocytes that affects the overall functioning of the heart (Wright and Ress, 1998). Subsequent studies examining cardiomyocyte apoptosis and the loss of cell volume showed that volume-sensitive ion channels played a role in AVD (D’Anglemont de Tassigny et al., 2004; Wang et al., 2005b; Okada et al., 2006). Specifically, volume-sensitive chloride channels (I_Cl,vol_) known to play a role in the regulation of cell volume (RVD), were also shown to be active during AVD (D’Anglemont de Tassigny et al., 2004; Figure 2). In adult rabbit ventricular cardiomyocyte treated with doxorubicin and C(2)-ceramide to initiate cell death, AVD and apoptosis were abolished upon exposure to the I_Cl,vol_ inhibitors 5-nitro-2-(3-phenylpropylamino) benzoic acid (NPPB) or indanyloxyacetic acid 94 (IAA-94; D’Anglemont de Tassigny et al., 2004; Figure 2). Additionally, volume-sensitive outwardly rectifying (VSOR) chloride channels were shown to be involved in staurosporine treated primary mouse ventricular myocytes (Okada et al., 2006) and neonatal rat cardiomyocytes (Liu et al., 2013) undergoing apoptosis. Recently, inhibition of (VOSR) chloride channels were shown to improve cardiac contractility and survivability in a turnicamycin-induced cardiomyocyte ER stress model (Shen et al., 2014). In a follow-up study, Wang et al. (2017) showed during high glucose-induced apoptosis, chloride channel blockers DIDS and DCPIB prevented activation of (VSOR) chloride channels and improved the viability of cardiomyocytes.

What is noteworthy about the volume-sensitive channels during AVD is their activation in the absence of cell swelling. While the precise nature of the isotonic activation of volume-sensitive channels is unknown, Okada et al. (2006) hypothesized several mechanisms that may permit channel activity including a lower threshold for the channel volume set point in apoptotic cells, the presence of reactive oxygen species, kinase activation and/or changes in the level of ATP as plausible mechanisms for channel activation. Subsequently, Liu et al. (2013) showed that PI3K/Akt played a major role in the activation of (VSOR) chloride channels during staurosporine-induced cardiomyocyte death (Figure 2). Additionally, Shen et al. (2014) suggested the involvement of the C/EBP homologous protein CHOP and Wnt inactivation upon ER stress in cardiomyocytes. Regardless of the precise mechanism, it is well-established that volume-sensitive ion channels become activated during cardiomyocyte apoptosis and result in the concomitant egress of water from the cells and persistent cell shrinkage defined as AVD.

Interestingly, beside studies examining the role of chloride channels during cardiomyocyte AVD, little information exists on other ionic flux that may be involved during apoptosis. In an oxidative stress model, cardiomyocytes treated with H_2_O_2_ showed a marked increase in intracellular sodium and calcium via reverse mode of the Na–Ca exchanger resulting in apoptosis (Yang et al., 2004; Figure 2). Interestingly, apoptosis was completely prevented under sodium-free conditions, but not calcium-free. Additionally, apoptosis occurred when a sodium ionophore cocktail in calcium-free medium was used instead of H_2_O_2_, suggesting the increase in intracellular sodium alone can signal the programmed cell death process in cardiomyocytes.

Glucocorticoids have been a classical apoptotic agent from the very first reports defining this physiological programmed cell death process. Intriguingly, treatment of cardiomyocytes with the synthetic glucocorticoid dexamethasone resulted in cardiac hypertrophy, and protected these cells from both serum deprivation and TNFα-induced apoptosis (Ren et al., 2012). Earlier studies showed that dexamethasone inhibited serum deprivation- and UV-C-induced apoptosis in rat hepatoma cells (Evans-Storms and Cidlowski, 2000; Scoltock et al., 2006). Interestingly in the latter study (Scoltock et al., 2006), dexamethasone did not prevent anti-Fas-induced apoptosis in hepatoma cells, suggesting the protective nature of glucocorticoids may be specific to agents that activate the intrinsic, and not the extrinsic apoptotic process.

## Corneal Epithelial Cells and AVD

The corneal epithelium, the outermost layer of the cornea composed of several layers of non-keratinized, stratified squamous epithelia cells that covers the front of the cornea, is shielded by a protective tear film consisting of an electrolyte- and protein-rich aqueous-mucus layer. The cornea functions to protect the intraocular contents of the eye along with serving as the principal optical element allowing formation of an image on the retina. Precise maintenance of electrolytes forming the osmotic gradient between the tear film and the ocular surface epithelia is important for cellular function and homeostasis. The ionic composition of tear film has been established *in situ* along with showing a critical role for aquaporins (water channels) in maintaining osmotically driven water transport across the cornea and tear film layer (Levin and Verkman, 2004; Ruiz-Ederra et al., 2009; Woodward et al., 2012). An imbalance of electrolytes in the tear film layer is a hallmark of many ocular diseases, most notably dry eye. Thus, the corneal epithelium is the main cellular barrier between the external environment and the operative components of the visual system.

As the osmotic gradient between the tear film and the ocular surface cell epithelia is vital, corneal epithelial cells are known to have various inherent volume regulatory mechanisms (Al-Nakkashj et al., 2004; Capó-Aponte et al., 2005, 2007). Similar to other cells in the body, volume regulatory mechanisms in corneal epithelial cells are comprised of analogous channels and transporters namely volume-regulated anion channels, potassium channels, the K–Cl and Na–K–Cl cotransporters, and the Na–K-ATPase. Additionally, channels such as the non-selective cation channel (Transient Receptor Potential Vanilloid 4; TRPV4) have been shown to have a role in RVD, as a decrease in TRPV4 expression and activity in corneal epithelial cells suppresses RVD (Pan et al., 2008).

Along with various channels and transporters involved in volume regulatory responses, kinase signaling pathways have also been shown to have a critical role. In a swelling-induced model of rabbit corneal epithelial cells, activation of extracellular signal-regulated kinase (ERK) and stress-activated protein kinase/c-Jun N-terminal kinase (SAPK/JNK) preceded both chloride and potassium channel activity in the RVD response (Pan et al., 2007). During hyperosmotic stress-induced corneal epithelial cell death (an *in vitro* dry eye model), activation of Polo-like kinase 3 (Plk3) and c-Jun were observed to promote the cell death program (Wang et al., 2011). In a recent study, KIOM-2015EW (a hot water extract of maple leaves) was shown it could protect the ocular surface from hyperosmolar stress in part by inhibiting MAPK kinase signaling (Kim et al., 2018).

While corneal epithelial cells have inherent cell volume regulatory mechanisms, they are still at risk of environmental insults. From exposure to fresh water while swimming, use of hypotonic eye drops, or pathological conditions such as dry eye disease, corneal epithelial cells can experience periods of persistent hypotonic or hypertonic stress that can lead to cell death. Additionally, other events can also have a negative impact on the eyes ranging from not eating a well-balanced diet to too much time in front of a computer and/or cellphone screen. Furthermore, while most people are aware of the harmful effects of UV light on the skin, few consider the equaling damaging effects excessive UV light has on the eyes. The corneal epithelium is continually exposed to ambient outdoor UVB and UVA. Therefore, it is not surprising that many diseases of the eye can be traced back to excessive UV light including cataracts, macular degeneration, and cancer of the eye.

It has been known for over 20 years that UV exposure results in corneal epithelial cell apoptosis (Podslochy et al., 2000; Lu et al., 2003). What also became apparent very early was the role potassium channels played in corneal epithelial cell death (Wang et al., 2003, 2005a). UV irradiation resulted in corneal epithelial cell apoptosis through the hyperactivation of potassium channels in the cell membrane (Lu, 2006), and inhibition of potassium channels with specific potassium channel blockers resulted in a reduction of numerous apoptotic characteristics including caspase activity, and DNA degradation (Ubels et al., 2010, 2011; Glupker et al., 2016). The opening of potassium channels leads to a rapid loss of intracellular potassium, fast cell shrinkage, and consequentially the activation of stress-related signaling pathways including the c-Jun N-terminal kinase cascade and p53 activation. Not surprisingly, elevated extracellular potassium was shown to prevent apoptosis in UV-B exposed corneal epithelial cells (Singleton et al., 2009), including reducing caspase activity (Leerar et al., 2016). Additionally, in this study the authors showed that caspase-9 had little activation, while caspase-8 was activated upon UV-B exposure, suggesting a major route of apoptotic induction was through the extrinsic pathway. However, a latter study by Ubels et al. (2016) suggested that the intrinsic apoptotic pathway is the major contributor to UVB-induced apoptosis in human corneal limbal epithelial cells, but TNF-R1 and FADD pathway still played an integral part in the UVB-induced potassium efflux (Boersma et al., 2017). What was clear from these and other studies where high extracellular potassium was shown to prevent the deleterious effects of UV exposure on corneal epithelial cells (Schotanus et al., 2011; Ubels et al., 2011) is that the relatively high concentration of potassium in tears works to suppress the loss of potassium from corneal epithelial cells in response to UV exposure. Therefore, tears function as a defensive measure in protecting the ocular surface not only from changes in extracellular osmolality, but also from ambient UV radiation.

Finally, elevated tear osmolarity exposes corneal epithelial cells to extracellular osmotic stress; a key pathological factor in dry eye disease. To combat the loss of cell volume or cell shrinkage as a result of this hypertonic stress on corneal epithelial cells, organic osmolytes such as betaine that can act as an osmo-protectant have been studied (Garrett et al., 2013). The presence of betaine in hypertonic-stressed human corneal limbal epithelial cells reduced the loss of cell volume from 27 to 11%, reduced caspase-8,-9, and -3/7 activity, and increased cell viability, suggesting agents such as betaine stabilized corneal epithelial cell volume under hyperosmotic stress, thus limiting the extent of apoptosis.

## Perspectives

The morphological loss of cell volume or AVD is a defining characteristic of apoptosis and suggests it plays a critical role during cell death. Whether it’s simply to decrease the size of cells to be easily phagocytized by resident macrophage and/or neighboring cells, or as an essential component of the apoptotic machinery, AVD is unique to this mode of programmed cell death. While many questions remain as to the role AVD plays during apoptosis, what has become apparently clear from studies on AVD and water movement in less common model systems is the existence of cell-type specific mechanisms for apoptosis. The extension of apoptotic studies in models such as neuronal cells, cardiomyocytes, and corneal epithelial cells has illustrated how individual cells employ their endogenous channels, transporters, and/or exchangers to carry out the AVD process and aids our understanding of how ionic and water flux relate to the execution of apoptosis. Finally, additional information gained from studying AVD in diverse model systems extends our knowledge of cell death and the role it plays in human disease.

## Author Contributions

CB and JC wrote the review. Both authors contributed to the article and approved the submitted version.

## Conflict of Interest

The authors declare that the research was conducted in the absence of any commercial or financial relationships that could be construed as a potential conflict of interest.
